# Letter to the editor: Response to Giardiello D, Antoniou AC, Mariani L, Easton DF, Steyerberg EW

**DOI:** 10.1186/s13058-020-01274-x

**Published:** 2020-04-10

**Authors:** Chang Ming, Valeria Viassolo, Nicole Probst-Hensch, Pierre O. Chappuis, Ivo D. Dinov, Maria C. Katapodi

**Affiliations:** 1 Department of Clinical Research, Faculty of Medicine, University of Basel, Missionstrasse 64, 2 OG – Room 007, 4055 Basel, Switzerland; 2 Oncogenetics and Cancer Prevention, Geneva University Hospitals, Geneva, Switzerland; 3 Swiss Tropical and Public Health Institute, University of Basel, Basel, Switzerland; 4 Genetic Medicine, Geneva University Hospitals, Geneva, Switzerland; 5 Department of Computational Medicine and Bioinformatics, University of Michigan, Ann Arbor, MI USA; 6 Michigan Institute for Data Science, University of Michigan, Ann Arbor, MI USA; 7 Statistics Online Computational resource, University of Michigan, Ann Arbor, MI USA; 8 University of Michigan School of Nursing, Ann Arbor, MI USA

We appreciate the opportunity to submit a response to the Letter to the Editor by Giardiello and colleagues [1] addressing our publication in Breast Cancer Research [2].

Giardiello and colleagues mentioned that our machine learning (ML) models were not specific for survival data. BCRAT/BOADICEA were developed and validated using survival data with binary outcomes and retrospective case control/cross-sectional data, respectively [3]. Their clinical application requires only cross-sectional data. Our ML models included the same risk factors and data structure in each comparison as BCRAT/BOADICEA. To avoid exaggerating the function of ML models, we generated the probability of whether a woman at a given age would develop breast cancer in her life, and not specific time frame risks (5-year or 10-year risk).

Giardiello and colleagues mentioned that our validation was unfair because we applied only internal validation processes. Cross-validation is not equivalent to internal validation; it is a statistical out-of-sample testing technique, which pools the results across many iterations, while each fold and each iteration do not blend training and testing data. A slight bias (aka surrogate problem) occurs because the cross-validation training sets are smaller than the original dataset. A 10-fold cross-validation relies on training sets that include 90% of the original dataset. In our study, this translated into two considerable sample sizes, *n*_1_ = 1029 from the US population-based data and *n*_2_ = 2233 from the Swiss clinic-based data. This lower-sample-size bias often translates into more conservative fit/prediction estimates [4].

Giardiello and colleagues mentioned that a fair comparison of the final models requires reporting parameter estimates and calibration. Reporting parameter estimates and their confidence intervals in the final model is not always possible [5]. We generated 80 parameter estimates for each risk factor based on different ML algorithms and different cross-validation summary approaches. The interpretation and usefulness of these estimates for each risk factor is limited without reference values from BCRAT/BOADICEA. Moreover, better/worse calibration does not lead to better/worse class-based or probability-based predictions [6]. Calibration comparisons was not our aim. ML may generate “aggressive” prediction calibration for minor classes due to “increased” sample size through rebalancing processes. Several recalibration methods can be applied and significantly improve some of the ML calibrations and predicted probabilities [6], making calibration comparisons of ML to BRCAT/BOADICEA unfair. Calibrated predicted probabilities should also fit clinically meaningful sensitivity and specificity for patient stratification, instead of one cutoff (cancer/no cancer) [7].

A prediction model cannot be developed, validated, and tested for utility at once. However, the development and validation of our ML models improved model predictive accuracy efficiently, i.e., using less time and fewer resources. Investing into promising new analytic approaches would improve research in the field of disease prediction and significantly further our knowledge about the potential application of ML in personalized medicine.

## Data Availability

Not applicable.
